# South African caregivers of children with autism during COVID-19: A scoping review

**DOI:** 10.4102/sajcd.v71i1.1017

**Published:** 2024-07-18

**Authors:** Jade Berson, Skye N. Adams

**Affiliations:** 1 Department of Speech Pathology and Audiology, School of Human and Community Development, University of the Witwatersrand, Johannesburg, South Africa

**Keywords:** autism, COVID-19, experiences, scoping review, service provision

## Abstract

**Background:**

The coronavirus disease 2019 (COVID-19) outbreak has had a profoundly negative impact on people all over the world, particularly those with disabilities such as autism. However, there are gaps in research understanding the impact of COVID-19 on this population and the support required.

**Aim:**

To explore the evidence available on the impact of the COVID-19 pandemic on caregivers of children with autism.

**Method:**

A scoping review methodology using the Arksey and O’Malley framework was employed. Five electronic databases from March 2020 to December 2022 were reviewed. Two thousand two hundred and six articles were retrieved with primary search terms: caregivers (population), autism (diagnosis) and COVID-19 (context). Following the screening of titles, abstracts and articles, 36 articles were included in the final review. Thematic and content qualitative analysis was completed.

**Results:**

Preferred reporting items for systematic reviews and meta-analyses extension for scoping reviews (PRISMA-ScR) guided the reporting of the findings. Three main themes were identified: (1) caregiver’s mental health and wellbeing, (2) response to remote health care and support and (3) caregiver resilience.

**Conclusion:**

The pandemic affected children with autism and their families regarding changes in routine, difficulties with support and emotional states. However, little research has been conducted on the impact in upper-middle-income countries such as South Africa.

**Contribution:**

The findings from this review carry practical implications that extend beyond the pandemic, such as political instability or natural disasters that may present similar stressors for children with autism and their families.

## Introduction

The emergence of the SARS-CoV-2 virus, responsible for COVID-19, was declared a global health emergency, profoundly impacting individuals worldwide and affecting their health, wellbeing, and financial stability (Al Naamani et al., 2020; Jeste et al., 2020; Laborde, 2020; Pellicano & Stears, 2020; The Lancet, 2020). The pandemic led to widespread lockdowns and restrictions, resulting in social and economic disruptions across countries. Coronavirus disease 19 disrupted daily life worldwide, affecting health care systems, economies and people’s overall quality of life (Chenneville & Schwartz-Mette, 2020; Hammons & Robart, 2021). Extensive research has emerged to understand the comprehensive impacts of the pandemic, revealing that certain populations, such as those with neurodevelopmental disabilities such as autism and their families, were particularly vulnerable to adverse effects from COVID-19. Children with autism and their families experienced disruptions to daily life, education, mental health, access to support services and overall wellbeing (Amaral & De Vries, 2020; Baweja et al., 2022; Bellomo et al., 2020; Nollace et al., 2020). The intersectionality of the pandemic’s effects on public health, socio-economic factors and health care delivery underscores the necessity for a scoping review.

Children with autism, who already face unique challenges in communication and socialisation, were particularly vulnerable during the unprecedented global health crisis. Many children with autism present with difficulties in social interaction, communication, sensory processing and adaptive behaviours requiring tailored support and intervention (Malcolm-Smith et al., 2013; McStay et al., 2014; Schaaf et al., 2014). Because of the characteristics of autism, this can be a source of stress and anxiety for caregivers (Adams et al., 2020; Ausderau et al., 2019; Lai & Oei, 2014). Research has demonstrated that children with autism and their families have been significantly affected by COVID-19, experiencing additional and exacerbating challenges in their development such as developmental delays and regressions, mental health and caregiver wellbeing because of social isolation and reduced access to essential services (Adams et al., 2022; Amaral & De Vries, 2020; Bellomo et al., 2020; Cassidy et al., 2020; Lugo-Marín et al., 2021).

Children with autism and their caregivers faced unique trials during the pandemic, particularly those who continued to work from home and care for their children. The disruptions caused by COVID-19 affected service delivery, support services and therapies that children with autism require for their development and communication skills (Baweja et al., 2022; Johnsson & Bulkeley, 2021). Many children with autism require routine and structure (Marquenie et al., 2011). However, a loss of this led to increased behavioural issues and difficulties for both children and caregivers (Adams et al., 2022; Baweja et al., 2022). Additionally, the pandemic created problems with both eating and sleeping patterns in children with autism because of changes to their routines and diets, leading to further challenges for caregivers (Friesen et al., 2022; Lee et al., 2021). Financial insecurity and job loss have been prevalent among families with children with autism, adding to the stress and burden experienced by caregivers.

The pandemic highlighted the importance of understanding and providing support to children with autism and their caregivers, recognising that their challenges are not confined to the pandemic but persist as ongoing concerns requiring sustained attention and resources. Research from various countries has shown that caregivers globally in comparison to individuals have experienced poor mental health, stress and fear during the pandemic because of intensified caregiving responsibilities, reduced support networks to assist with childcare and the lack of personal time (Friesen et al., 2022; Lee et al., 2021; Lugo-Marín et al., 2021). It is crucial to address the unique requirements of children with autism and their caregivers, both during and after the pandemic, to ensure their wellbeing and development. The experiences of caregivers during the pandemic provide valuable insights into the challenges faced and the support required during times of crisis and beyond. Understanding the specific impact of COVID-19 on children with autism is crucial for developing targeted interventions and support strategies to mitigate the negative consequences. Therefore, this scoping review aims to identify the challenges faced by caregivers of children with autism during the COVID-19 pandemic and to consolidate strategies and supports required for these families as they are the main stakeholders for their children.

## Methods

The scoping review aimed to systematically identify and analyse available research on the experiences caregivers of children with autism had during the COVID-19 pandemic. Researchers Arksey and O’Malley (2005) characterise a scoping review as a method for systematically mapping the extent, range and nature of relevant literature within a specific field of interest. Therefore, the scoping review was identified as an appropriate methodology to meet the aims of this study. This framework involves five key stages: (1) identifying the research question, (2) identifying relevant studies, (3) study selection, (4) charting the data and (5) collating, summarising and reporting the results used to conduct the scoping review.

### Data sources and search strategy

A systematic electronic search for articles was conducted between May and July 2022. Four databases were used: ScienceDirect, PubMed, Sage Journals and BMJ Journals. The databases were chosen to be comprehensive and to cover diagnosis and autism. The cut-off date for the electronic search was articles published between March 2020 and December 2022, aligning with the time frame of the pandemic and subsequent publication of research articles. The search strategies were drafted in consultation with an experienced librarian and further refined and reviewed by the two authors (J.B. and S.N.A.). Keywords were generated and related to concepts around parenting, autism and COVID-19. Because of the novelty and scarcity of COVID-19 literature available, the search terms remained broad. This search strategy was applied to all four databases. BOOLEAN operators (AND and OR) as well as truncation were used in the search strings using subject headings (MeSH), and keywords with proximity operators, respectively (Table 1). Subject headings were further refined to optimise the search results for each database. A hand search of the reference and citation lists was also conducted to locate additional eligible studies.

**TABLE 1:** Rapid review literature search terms.

Criteria	Research database terms
Target population	Family, parent and caregiver
Diagnosis	Autism spectrum disorder, autism, autistic and developmental disability
Context	COVID-19 and coronavirus

### Study selection

The current review included published peer-reviewed journal articles, was limited to primary study designs (qualitative, quantitative and mixed-method approaches), and excluded grey literature and non-academic sources. Grey literature and non-academic sources were excluded to ensure the use of high-quality, reliable and peer-reviewed academic sources, maintaining focus and reproducibility within the constraints of time and resources. Articles were included if they had information on parental experiences with their children with autism during COVID-19. The following inclusion criteria were used: (1) studies presented original research published in a peer-reviewed journal, (2) study participants included primary caregivers of individuals with autism (e.g. mothers, fathers, foster parents and grandparents), (3) the topic of the study clearly focused on the impact of COVID-19, (4) published between 2020 and 2022, (5) articles published in English and (6) only primary studies were included. Studies including participants with unspecified diagnostic groups (e.g. a sample of individuals with unspecified developmental and/or intellectual disabilities) were excluded to provide a homogenous evidence base as well as studies relating to adults over 18 with autism and those focusing specifically on COVID-19 with no mention of autism.

The search was undertaken by the first reviewer (J.B.) and Mendeley software was used to import articles and remove any duplicates. All titles and abstracts were screened for relevance by two reviewers independently (J.B. and S.N.A.) and were then assessed for full-text eligibility before being included in the final review. Any discrepancies noted in the screening process were resolved through discussion and consensus among both authors. An iterative approach to study screening and selection was employed to emphasise a more inclusive final list of studies. All articles were accessed electronically.

### Charting the data

The data charting template was created by J.B. and refined by S.N.A. Information extracted from the included studies: (1) caregiver and child characteristics (i.e. relationship to child and age of caregiver and child, all types of family structures were included); (2) country; (3) study design, method and data analysis and (4) key findings related to caregiver challenges and supports were collated and charted onto a document (Table 2). The initial draft of the chart underwent a pilot test using three randomly selected articles, independently reviewed by each author (J.B. and S.N.A). Following minor revisions based on the pilot test feedback, the finalised chart was then used independently by both reviewers. The agreement between the two reviewers was 100% during the screening process. Any disagreements were resolved by consensus. As a scoping review methodology is iterative, this allowed for an adjustment to be made regarding the inclusion of relevant studies during consensus discussions (Levac et al., 2010).

**TABLE 2:** Characteristics of included studies (*N* = 36).

Article number	Author(s)/ title of article	Participants	Method of data collection	Country	Children characteristics	Caregiver characteristic
1	Althiab (2021)	211 caregivers	Questionnaire	Saudi Arabia	Age in years (*N*, %) 3–7: 57 (27) 8–12: 119 (56.4) 13–17: 35 (16.6) Male (*N*, %): 164 (77.7) Female (*N*, %): 47 (22.3)	Age in years (M, s.d.): 34.7 (6.8) Male (*N*, %): 84 (39.8) Female (*N*, %): 127 (60.2)
2	Amirova et al. (2022)	97 caregivers	Mixed methods Online survey and interviews	Kazakhstan	Age in years (*N*, %) 5 and below: 36 (40) 6–8: 36 (40) 9 and above: 18 (20) Male (*N*, %): 65 (70.7) Female (*N*, %): 27 (29.3)	Age in years (*N*, %) 30 and below: 22 (22.7) 30–393%): 2 (2) 40 and up: 21 (21.6) Male (*N*, %): 2 (2) Female (*N*, %): 95 (98)
3	Arazi et al. (2022)	268 caregivers	Online survey	Israel	Age in years (M, s.d.): 7.6 (4.3) Male (*N*, %): 215 (80) Female (*N*, %): 53 (20)	Age in years (M, s.d.): 41 (6.4)
4	Athbah (2021)	217 caregivers	Questionnaire	Saudi Arabia	Male (*N*, %): 138 (63.9) Female (*N*, %): 78 (236.1)	
5	Azevedo Machado et al. (2022)	721 caregivers	Online survey	Brazil	Age in years (range): 3–18 Severity: Level 1: 432 (43) Level 2: 481 (48) Level 3: 88 (9)	
6	Bhat (2021)	6396 caregivers	Survey	United States (US)	Age in years: 3 and below: 89 (1.4) 3–9: 2686 (42) 9–15: 2382 (37) 15–18: 937 (14.7) Male (*N*, %): 5158 (80.7) Female (*N*, %): 1235 (19.3)	Ethnicity (*N*, %): White people 4191 (65.6) Asian people: 104 (1.6) Black people: 248 (3.9) Native American: 20 (0.31) Native Hawaiian: 3 (0.05) Hispanic people: 6 (0.09) Not specified: 264 (4.1)
7	Bozkus-Genc and Sani-Bozkurt (2022)	8 caregivers	Semi-structured interviews	Turkey	Male (*N*, %): 7 (12.5) Female (*N*, %): 1 (87.5) Severity (*N*, %): Level 1: 4 (50%) Level 2: 2 (25%) Level 3: 2 (25%)	Age in years (M, range): 46.4 Male (*N*, %): 1 (87.5) Female (*N*, %): 7 (12.5)
8	Chen et al. (2021)	1450 caregivers (454 caregivers for children with autism)	Online survey	China	Age in years (M, s.d.): 11.38 (3.34)	Age in years (M, s.d.): 40.76 (5.84) Male (*N*, %): 124 (27.31) Female (*N*, %): 330 (72.69)
9	Colizzi et al. (2020)	527 caregivers	Online survey	Italy	Age in years (M, s.d.): 13 (8.1) Comorbidity: Yes: 145 (27.8) No: 377 (72.2)	Male (*N*, %): 124 (27.31) Female (*N*, %): 330 (72.69)
10	Corbett et al. (2021)	122 mothers (61 mothers of children with autism)	Longitudinal study	US	Age in years (M, s.d.): 13.23 (1.16) Severity ADOS (M, s.d.) 7.13 (2.03)	Race: White people (84.42%) Black people (4.92) Mixed Race (10.66)
11	Cusinato et al. (2020)	463 caregivers	Online survey	Italy	Age in years (M, s.d.): 12.1 (5.44) Male (*N*, %): 260 (56.2) Female (*N*, %): 203 (43.8)	Age in years (M, s.d.): 43.3 (5.88) Male (*N*, %): 47 (9.5) Female (*N*, %): 416 (90.5)
12	Dekker et al. (2022)	27 caregivers	Semi-structured interviews and surveys	US	Age in years (range): 4–21	-
13	Yarımkaya & Esentürk (2020)	10 caregivers	Semi-structured interviews	Turkey	Age in years (M, range): 11.2 (9–16) Male (*N*, %): 5 (50) Female (*N*, %): 5 (50)	Age in years (M, range): 42 (36–54) Male (*N*, %): 4 (40) Female (*N*, %): 6 (60)
14	Fong et al. (2021)	72 caregivers	Online survey	Malaysia	Age in years (M, s.d.): 9 (2.91) Male (*N*, %): 54 (75) Female (*N*, %): 18 (25) Severity: Level 1: 38 (51.4) Level 2: 23 (31.9) Level 3: (5.6) Unspecified: 7 (9.7)	Age in years (M, s.d.): 38.3 (5.62) Male (*N*, %): 18 (25) Female (*N*, %): 54 (75) Ethnicity (*N*, %): Malay: 55 (76.4%) Chinese: 6 (8.3) Indian: 5 (6.9) Other: 6 (8.3)
15	Huang et al. (2021)	406 caregivers	Online survey	China	Age in years (M, s.d.) 4.6 (2.3) Male (*N*, %): 331 (81.5) Female (*N*, %): 75 (18.5)	
16	Iovino et al. (2021)	337 caregivers	Survey	US	Age in years (range): 6–18 Male (*N*, %): 133 (70.4) Female (*N*, %): 55 (29.1)	Age in years (*N*, %): 18–34: 13 (6.8) 35–54: 172 (91) 65 and up: 2 (1.1) Male (*N*, %): 83 (43.9) Female (*N*, %): 105 (55.6) Race (*N*, %): American-Indian: 4 (2.1) Asian people: 8 (4.2) Black people 23 (12.2) White people 150 (79.4)
17	Kaku et al. (2021)	153 caregivers	Online questionnaire	India	Age in years (range, %): 2–11 (70) 12–25 (30) Males (*N*, %): 125 (81.7) Females (*N*, %): 28 (18.3) Comorbidities: Yes: 94 (62) No: 59 (38)	
18	Kalb et al. (2021)	5506 caregivers	Cross-sectional study Online surveys	US	Age in years (M, s.d.): 10.2 (4.0) Male (*N*, %): 4436 (81) Female (*N*, %): 1070 (19)	Age in years (*N*, %): 18–29: 220 (4) 30–49: 4680 (85) 50 and up: 46 (15.4) Male (*N*, %): 191 (5) Female (*N*, %): 3365 (95) Race (*N*, %): Black people: 220 (4) Hispanic people: 936 (17) White people: 4130 (75)
19	Tokatly Latzer et al. (2021)	31 caregivers (25 children with autism)	Semi-structured telephonic interviews	Israel	Age in years (M): 5.11 Male (*N*, %): 22 (88) Female (*N*, %): 3 (12) Severity: Level 1: 9 (36) Level 2: 8 (32) Level 3: 8 (32)	Male (*N*, %): 6 (19) Female (*N*, %): 25 (81)
20	Khan et al. (2021)	58 caregivers	Semi-structured interview and survey	State of Qatar	Age in years (M): 14.8 Male (*N*, %): 48 (83) Female (*N*, %): 10 (17) Comorbidity (*N*, %): Yes: 32 (55.2) No: 26 (44.8)	
21	Levante et al. (2021)	120 caregivers (53 caregivers of children with autism)	Online survey	Italy	Age in years (M, s.d.): 6.94 (1.6) Male (*N*, %): 43 (81.1) Female (*N*, %): 10 (9.9) Severity: Low: 26 (49) High: 27 (51)	Age in years (M, s.d.): 41.8 (5.4) Male (*N*, %): 9 (17) Female (*N*, %): 44 (83)
22	Logrieco et al. (2022)	243 parents	Online survey	Italy	Age in years (M, s.d.): 7 (3.3) Male (*N*, %): 209 (86) Female (*N*, %): 34 (14) Severity (*N*, %): Level 1 66 (27.1) Level 2: 108 (44.5) Level 3: 69 (28.4)	Age in years (M, s.d.): 40.4 (7.05) Male (*N*, %): 20 (8.2) Female (*N*, %): 223 (91.8)
23	Lopata et al. (2022)	69 parents/caregivers	Online scales and checklist	Not stated	Age in years (M, s.d.): 12.32 (1.56) Male (*N*, %): 62 (90) Female (*N*, %): 7 (10)	Race (*N*, %): White people: 66 (96%) Others not specified
24	Lugo-Marín et al. (2021)	37 caregivers	Questionnaire	Spain	Age in years (M, s.d.): 10.7 (3.4) Male (*N*, %): 31 (86.5) Female (*N*, %): 6 (13.5) Severity (*N*, %): Level 1: 26 (70.3) Level 2: 11 (29.7) Level 3: 0 (0)	Age in years (M, s.d.): 42.5 (11.7) Male (*N*, %): 5 (13.5) Female (*N*, %): 32 (86.5)
25	Manning et al. (2021)	459 caregivers	Online survey	US	Age in years (M, s.d.): 11.8 (7.9) Under 21: 402 (86.6) Over 21: 62 (13.4) Severity (M, %): Level 1: 183 (38.9) Level 2: 223 (49.5) Level 3: 55 (11.7)	-
26	Morris et al. (2021)	176 caregivers	Online questionnaires	United Kingdom	Age in years (M, %): 3–4: 17 (9.7) 5–6: 32 (18.2) 7–8: 42 (23.9) 9–10: 46 (26.1) 11–12 39 (22.2)	-
27	Mumbardó-Adam et al. (2021)	47 caregivers	Online questionnaire	Spain	Age in years (M, s.d.): 7.3 (3.4) Male (*N*, %): 36 (76.6) Female (*N*, %): 11 (24.4)	Age in years (M,s.d.): 41.3 (6.2) Male (*N*, %): 9 (19.1) Female (*N*, %): 38 (80)
28	Panjwani et al. (2021)	200 caregivers	Online survey	US	Age in years (M, s.d.): 7.7 (4.1) Male (*N*, %): 150 (76.1) Female (*N*, %): 47 (23.9)	Race: White people: 121 (62.1) Black people: 14 (7.2) Hispanic or Latino people: 27 (13.9) Mixed-race people: 19 (9.7) Other: 14 (7.2)
29	Papanikolaou et al. (2022)	62 caregivers	Questionnaire	Greece	Age in years (M, s.d.): 17.3 (3.3) Male (*N*, %): 44 (78.6) Female (*N*, %): 12 (21.4)	Age in years (M, s.d.): 49.6 (7.8) Male (*N*, %): 6 (10.7) Female (*N*, %): 50 (89.3)
30	Pecor et al. (2021)	575 caregivers (170 caregivers for children with autism)	Online questionnaire	US		Age in years (M, range): 50.3 (26–69) Male (*N*, %): 16 (9) Female (*N*, %): 154 (91)
31	Pellicano et al. (2022)	84 caregivers (35 parents of children with autism)	Qualitative study Semi-structured interviews	Australia	Age in years (M, s.d.): 10.21 (4.04) Male (*N*, %): 30 (54) Female (*N*, %): 22 (40)	Age in years (M, s.d.): 39.10 (11.50) Male (*N*, %): 32 (91) Female (*N*, %): 2 (6)
32	Polónyiová et al. (2022)	155 caregivers	Online questionnaire	Slovakia	Age in years (M, s.d.): 8.73 (3.64) Male (*N*, %): 113 (73) Female (*N*, %): 42 (27)	Age in years (M, s.d.): 39.38 (6.85)
33	Pratesi et al. (2021)	881 caregivers	Questionnaire	Brazil	Age in years (*N*, %): 5 and below: 412 (47) 6–11: 339 (39) 12 and up: 125 (14)	Age in years (*N*, %) 30 and under: 187 (21) 31–40: 475 (54) 41 and up: 213 (25) Male (*N*, %): 24 (3) Female (*N*, %): 857 (97)
34	Siracusano et al. (2021)	85 caregivers and 85 children with autism	Observational study	Italy	Age in years (M, range): 7 (2–18) Male (*N*, %): 68 (80) Female (*N*, %): 17 (20)	
35	Stankovic et al. (2022)	85 caregivers	Electronic survey	Serbia	Age in years (M, s.d.): 9.2 (4.5) Comorbidity (%): 47.7%	Family member (%): Mothers (74) Fathers (22) Other relatives and foster parents (4)
36	White et al. (2021)	3502 caregivers	Questionnaire	US	Age in years (M, s.d.): 11.8 (6.6) Male (*N*, %): 2797 (80) Female (*N*, %): 705 (20)	Age in years (M, s.d.): 43.4 (8.8) Male (*N*, %): 234 (7) Female (*N*, %): 3268 (93) Race (*N*, %): White people 2599 (80) Black people 145 (4) Asian people 74 (2) Native-American people 13 (0) Other 98 (3)

s.d., standard deviation; M, mean.

### Collating, summarising and reporting the results

After extraction, the findings from the included studies were separated, grouped, abstracted and categorised into themes. Independent categorisation based on the study objectives, challenges and supports was conducted by the two reviewers (J.B. and S.N.A). A thematic analysis was conducted using Braun and Clark’s framework (Braun & Clarke, 2006). Two reviewers extracted information from each article, grouped and labelled findings, categorised themes and summarised general trends on the experiences of caregivers of children with autism during COVID-19. The authors developed a code book (Online Supplementary, Table 1) provided different codes (and descriptions), as well as relevant articles and the frequency of each code. Similar codes were then collated and organised into themes and sub-themes. Prominent themes from the reviewers were then selected, relabelled and finalised after a comprehensive review and discussion between both reviewers.

### Ethical considerations

This article followed all ethical standards for research without direct contact with human or animal subjects. An ethics waiver was obtained from the University of the Witwatersrand Human Research and Ethics Committee (Non-Medical) (waiver number STA_2022_15).

## Review findings

**FIGURE 1:**
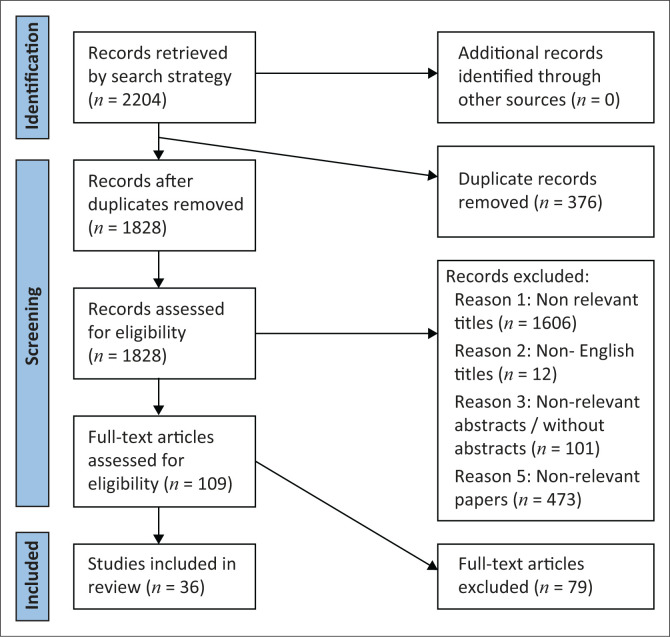
Preferred reporting items for systematic reviews and meta-analysis flow diagram for scoping reviews.

Results of the search strategy and study selection process are presented in a preferred reporting items for systematic reviews and meta-analyses extension for scoping reviews (PRISMA-ScR) (Moher et al., 2009) diagrammatic flow chart (Figure 1). The initial search yielded 2204 articles, and 376 duplicates were removed. No new articles were found through the review of the reference lists. Then, 1828 article titles and abstracts were screened for relevance and 1719 articles were excluded for the following reasons: different population focus, did not relate to COVID-19 and different diagnosis focus. The full texts of 109 articles were then screened for eligibility, and 79 were excluded leaving 36 articles included in the final review.

### Characteristics of the included studies

**FIGURE 2:**
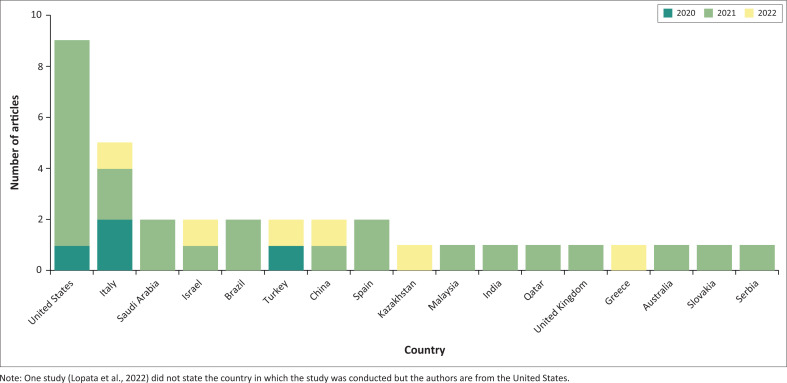
Number of articles and their publication dates from the identified countries.

Table 2 presents a summary of study characteristics and relevant data from the 36 studies included in the review. In this section, the study characteristics are described in more detail as follows: (1) study design and data collection methods (2) study locations and (3) study populations: (3.1) child characteristics and (3.2) caregiver characteristics:

- Study design and data collection methods
  There were different study methodologies employed with eight quantitative (22%), 21 qualitative (59%) and seven mixed-method designs (19%). Studies employed a diverse range of methods although most studies (86%) utilised online parent or caregiver surveys, such as online scales and questionnaires as their main form of data collection, primarily because of the restrictions imposed by the COVID-19 pandemic (Tremblay et al., 2021).
- Study locations
  Studies were conducted in 17 different countries across the globe. Figure 2 provides a summary of countries and articles. However, the majority were based in the United States (25.7%), followed by Italy (14.3%). This shows the absence of studies conducted in Africa with an increasing trend of publications from 2020 to 2022 with the majority of studies occurring during 2021 (72%). Although information from other upper-middle-income countries can still provide guidance for application in South Africa regarding response to future pandemics. The predominant focus of the studies was on the caregiving experiences of individuals with children with autism, delving into various facets such as parental mental health and the perceived impact on their children.
- Study populations
  ■ Children characteristics
    Across 36 studies, 30 studies focused on children between the ages of 2 and 18 years old (83%) and three studies went past the child and adolescent stage, including ages 18 and above (not specified) in their samples (8.3%) and the remainder of the studies (*n* = 3) did not report on the child’s age. Older children were included as many still lived at home and were dependant on the care provided by their caregivers. In addition, the children with autism included in the study were predominantly male, with only nine (25%) studies mentioning the level of support required for the child with autism (Azevedo Machado et al., 2022; Bozkus-Genc & Sani-Bozkurt, 2022; Corbett et al., 2021; Fong et al., 2021; Levante et al., 2021; Logrieco et al., 2022; Lugo-Marín et al., 2021; Manning et al., 2021; Tokatly Latzer et al. 2021). In addition, four (11%) studies made reference to associated comorbidities (Colizzi et al., 2020; Kaku et al., 2021; Khan et al., 2021; Stankovic et al., 2022).
  ■ Caregiver characteristics
    The majority of the caregivers in the studies were mothers (80%), reflecting the common role of mothers as primary caregivers in South Africa (Hatch & Posel, 2018). This aspect was specified in 21 (61%) of the studies. It was interesting to note that even when studies did recruit both mothers and fathers, mothers mainly completed the questionnaires or surveys. Eight of the included studies (22%) addressed the aspect of race, with the majority of the responding sample being white (60%). Six studies (17%) also included minority populations, specifically Hispanic and African American participants, which were documented across the research (Bhat 2021; Corbett et al., 2021; Iovino et al., 2021; Kalb et al., 2021; Panjwani et al., 2021; White et al., 2021).

### Qualitative findings

Selected studies were sorted into the three most prominent themes: (1) caregiver’s mental health and wellbeing, (2) response to remote health care and support and (3) caregiver resilience.

#### Caregiver’s mental health and wellbeing

Many caregivers highlighted the adverse impact of the COVID-19 pandemic on their psychological wellbeing, influenced by various factors related to restrictions and challenges with their children with autism. The code addressing mental health challenges surfaced in 23 studies (64%) (Althiabi, 2021; Arazi et al., 2022; Athbah, 2021; Azevedo Machado et al., 2021; Bhat, 2021; Chen et al., 2020; Dekker et al., 2022; Fong et al., 2021; Kaku et al., 2021; Kalb et al., 2021; Levante et al., 2021; Logrieco et al., 2022; Lugo Marin et al., 2021; Manning et al., 2021; Mumbardó-Adam et al., 2021; Papanikolaou et al., 2022; Pecor et al., 2021; Pellicano et al., 2022; Polionyiova et al., 2021; Pratesi et al., 2021; White et al., 2021). Caregivers reported heightened levels of stress, anxiety and depression because of the pandemic, noting that while some of these feelings existed before the pandemic, they were intensified by the additional stressors and imposed COVID-19 restrictions (Logrieco et al., 2022). Coronavirus disease 19 brought about drastic changes to daily routines and imposed social restrictions, which are particularly difficult for caregivers of children with autism, while also taking away supportive services such as schools and therapy with a lot of uncertainty as to when the restrictions would be lifted. This created a compounded effect on their mental health. In a study conducted in Saudi Arabia, 94% of caregivers of children with autism reported an escalation in their stress levels, and 78.7% acknowledged a detrimental impact on their emotional wellbeing because of the pandemic (Alhuzimi, 2021).

Caregivers’ mental health and wellbeing were impacted as a result of various factors, such as financial stress, reduced access to services and feelings of loneliness. Many caregivers experienced heightened pressure as they took on additional responsibilities in caring for their children without adequate support. The responsibility of caring for a child with autism intensified because of restricted access to educational and therapeutic services, a lack of support systems, disrupted routines and health concerns for both caregivers and their children (Mumbardó-Adam et al., 2021; Stankovic et al., 2022). Notably, while these studies addressed these challenges, they did not offer insight into whether stress levels varied based on the specific age of the child, family composition or whether they were single parents. Sleep patterns were disrupted for both caregivers and children because of routine shifts such as changes in bedtime and waking hours (Chen et al., 2020), leading to a decline in sleep quality for both the child and their caregiver. These changes in routine, associated with sleep disturbances, are closely correlated with diminished mental wellbeing and reduced patience. Moreover, disruptions extended to aspects such as eating and social interactions (Pellicano et al., 2022; Tokatly Latzer et al., 2021), adding to the strain caregivers faced. Because of the altered routines, many caregivers reported difficulties in managing mealtimes and structured activities or educational tasks for their children (Azevedo Machado et al., 2021; Colizzi et al., 2020; Esentürk, 2021).

An additional and significant stressor identified was the pervasive feeling of loneliness among caregivers. Changes to families’ daily routines, encompassing activities such as schooling, therapy and sleep intensified the sense of isolation (Chen et al., 2020; Huang et al., 2021; Logrieco et al., 2022; Lugo-Marín et al., 2021; Polónyiová et al., 2022; Stankovic et al., 2022). Online interactions proved insufficient in alleviating the nostalgia for the way life used to be (Pellicano et al., 2022). The apprehension of contracting COVID-19 further heightened caregivers’ stress, impeding their ability to engage with the outside world and fulfil essential obligations, such as grocery shopping (Amirova et al., 2022; Athbah, 2021; Azevedo Machado et al., 2021; Dekker et al., 2022; Lugo-Marín et al., 2021; Polónyiová et al., 2022). The increased fear of the virus was particularly associated with concerns about caregivers and their children becoming infected, exacerbated by challenges in accessing health care services (Manning et al., 2021). The unavailability of therapy and health care services compounded caregivers concerns, leading to feelings of hopelessness and depression (Logrieco et al., 2022). Caregivers were required to navigate the challenging roles of caregivers and educators, adapting to at-home learning in isolation and with limited to no support.

#### Response to remote health care and support

The code addressing the response to remote health care support or teletherapy surfaced in 21 studies (58%) (Amirova et al., 2022; Arazi et al., 2022; Athbah, 2021; Bhat, 2021; Bozkus-Genc & Sani-Bozkurt, 2022; Chen et al., 2021; Corbett et al., 2021; Dekker et al., 2022; Huang et al., 2021; Kaku et al., 2021; Kalb et al., 2021; Khan et al., 2021; Levante et al., 2021; Logrieco et al., 2022; Lopata et al., 2022; Manning et al., 2021; Papanikolaou et al., 2022; Pecor et al., 2021; Pellicano et al., 2022; Polónyiová et al., 2022; White et al., 2021). In response to the COVID-19 pandemic and restrictions on therapy, therapists globally turned to teletherapy as an alternative solution (Amirova et al., 2022; Bozkus-Genc & Sani-Bozkurt, 2022; Pellicano et al., 2022). While remote health care emerged as a significant theme in many articles, the widespread adoption of teletherapy faced substantial hurdles for the majority of children and their caregivers. Barriers such as the unavailability of online support and services, along with challenges related to the cost of data and Internet access, impeded teletherapy access. Approximately, 22% of participants received one or fewer consultations a week from teachers and therapists (Arazi et al., 2022; Logrieco et al., 2022; Manning et al., 2021).

While online resources were shared, only 20% of caregivers across studies found them helpful because of their limited relevance and their constraints in implementing caregiving knowledge (Arazi et al., 2022; Morris et al., 2021). Despite efforts, caregivers faced challenges with teletherapy, including difficulties in keeping their child in front of the screen (Arazi et al., 2022), along with problems related to digital competency, which hindered the implementation of teletherapy for caregivers already adapting to this new method of at-home learning or therapy. Caregivers perceived teletherapy as less engaging and supportive, with some discontinuing therapy altogether (Bozkus-Genc & Sani-Bozkurt, 2022).

Although some caregivers preferred in-person contact, others found teletherapy beneficial for the continuity of services and preventing regressions (Pellicano et al., 2022). An Italian survey indicated that teletherapy enhanced the caregiver’s quality of life during lockdown (Logrieco et al., 2022). However, the overall implementation of telehealth predominantly resulted in negative experiences across studies, compounding caregivers’ burden and affecting their emotional wellbeing and caregiving ability. It intensified their role in managing both their children and their daily tasks.

#### Caregiver resilience

Resilience emerged as a prominent theme in eight studies (22%) (Bozkus-Genc & Sani-Bozkurt, 2022; Colizzi et al., 2020; Cusinato et al., 2020; Kaku et al., 2021; Kalb et al., 2021; Lugo-Marín et al., 2021; Pellicano et al., 2022; Polónyiová et al., 2022). In the face of numerous challenges brought on by the COVID-19 pandemic, the ability to maintain positivity and resilience became indispensable for the optimal functioning of both caregivers and children. The exploration of resilience in these studies offers valuable insights that extend beyond the immediate context, providing a foundation for understanding and addressing future challenges and family support.

Based on the thematic analysis, parenting tips and suggestions for families with children with autism during challenging times to empower caregivers and their children were consolidated into five key points (Table 3).

**TABLE 3:** Parenting tips to support families with children with autism when access to services is challenged.

Parenting tip	Description	Relevant articles
Structure in daily routines	Implementing structured activities to ensure organised and routine-based task completion, promoting better coping for children during the pandemic. This approach allows caregivers to allocate time for self-care through intentional planning	Corbett et al. 2021, Iovino et al. 2021 and Morris et al. 2021
Positive outlook	A shift in perspective towards positivity as a strategy for caregivers to navigate their circumstances and capitalise on emerging positive aspects	Alhuzimi 2021, Logrieco et al. 2022 and Tokatly Latzer et al. 2021
Accessing remote counselling or health care services	Utilise remote services – if it is available. Access to mental health services for caregivers of children with autism can support emotions, negative psychological states and overall wellbeing during and after the pandemic	Bozkus-Genc & Sani-Bozkurt 2022, Iovino et al. 2021, Kalb et al. 2021, Polónyiová et al. 2022, Siracusano et al. 2021, Stankovic et al. 2022 and White et al. 2021
Online family support	Regularly scheduled phone calls or online communication methods within the family could offer valuable support to caregivers of children with autism	Althiabi 2021, Chen et al. 2020, Huang et al. 2021, Khan et al. 2021 and Siracusano et al., 2021
Family training	Seminars, online programmes or information sessions be held for caregivers to provide them with formal training	Althiabi 2021, Athbah 2021, Azevedo Machado et al. 2021, Cusinato et al. 2020, Kaku et al. 2021, Papanikolaou et al. 2022 and Pecor et al. 2021

Crafting structured routines emerged (*n* = 6, 17%) as a valuable mechanism for caregivers to effectively manage their child’s time at home and mitigate the risk of burnout during the COVID-19 pandemic (Corbett et al., 2021; Iovino et al., 2021; Kaku et al., 2021; Logrieco et al., 2022; Morris et al., 2021; Mumbardó-Adam et al., 2021). Specifically, structured play environments proved to be effective tools in navigating the challenges posed by the pandemic, facilitating learning and reducing caregiver stress. These environments empowered caregivers to manage and regulate their children, engaging them in purposeful and less overwhelming activities. The result was not only strengthened bonds between caregivers and children but also an improved understanding of the child’s needs within the home setting (Morris et al., 2021).

Maintaining a positive outlook emerged as a crucial factor associated with enhanced caregiver wellbeing and resilience, in contrast to feelings of denial and hopelessness (Tokatly Latzer et al., 2021). Caregivers not only required motivation but also practical tools to execute beneficial activities at home, bolstering their confidence. The presence of positive role models within households positively impacted the emotional states of both caregivers and children (Logrieco et al., 2022).

Children whose caregivers received online support through their community and engagement with social media during lockdown demonstrated improvements in self-care and environmental management, positively influencing the wellbeing of both the child and the caregiver (Siracusano et al., 2021). Several caregivers expressed a desire for support, yet remained uncertain about what measures could truly provide comprehensive assistance during such an unprecedented period (Colizzi et al., 2020). Caregivers expressed a need for consistent services, including psychological assistance (Logrieco et al., 2022; Pellicano et al., 2022; White et al., 2021). Regularly engaging in video conversations with family, friends and colleagues, some parents also received assistance from psychological therapists (Alhuzimi, 2021). Studies underscored the demand for enhanced health care and education services tailored to children and caregivers (Amirova et al., 2022; Arazi et al., 2022; Lopata et al., 2022). Iovino et al. (2021) underscored the importance of mental health support and policies tailored to caregivers in this demographic, aiming to alleviate caregiver burden and distress while promoting caregiver resilience both during and after the pandemic.

## Discussion

This scoping review provides a summary of evidence about the challenges faced by caregivers of children with autism during the COVID-19 pandemic and consolidates parenting strategies and supports required for these families. Previous reviews focused on caregivers’ experiences in general, examining the broader aspects of their roles and challenges. In contrast, this study specifically concentrates on their experiences during the COVID-19 pandemic and the distinctive challenges or events that characterised this particular period in their lives. During COVID-19, additional pressures were placed on caregivers of children with autism who were required to take on additional roles and responsibilities that went beyond caregiving to include support for therapy, schooling and prevention of regressions, often with limited or no support from teachers or health care professionals (Amirova et al., 2022; Arazi et al., 2022; Papanikolaou et al., 2022). As a result, caregivers experienced increased pressures and feelings of anxiety and mental health problems. The unique context of the pandemic provides insights that could be used to improve the general caregiving landscape, emphasising the need for stability and adaptability in services.

The COVID-19 pandemic led to a surge in pandemic-related research globally. Research on the experiences of caregivers of children with autism during COVID-19 has been expanding alongside autism awareness, with a growing number of studies utilising diverse methodologies across contexts. The majority of these studies, however, have been conducted in high-income settings, notably in the United States and Italy. While these studies contribute valuable insights into the effects of the pandemic on caregivers of children with autism, it is crucial to note the limited representation of research from Africa, specifically South Africa, highlights a critical gap. However, research from other low-middle and middle-upper-income countries can be used to inform the ways in which South Africa needs to consider continued support for children with autism and their caregivers both post-pandemic and beyond.

The skewed distribution of research towards high-income countries and participants is a notable limitation, primarily driven by the necessity for online survey distribution during the pandemic restrictions (Iovino et al., 2021; Ramlagan et al., 2022). This online-centric approach may have inadvertently excluded certain groups, particularly those who lack access to social media or online platforms, emphasising the importance of conducting research that employs methodologies contextually appropriate for diverse populations (Aderinto et al., 2023; Guler et al., 2023). Understanding the unique challenges faced by caregivers in Africa is essential for tailoring interventions and support systems that are culturally and contextually relevant, ultimately contributing to a more comprehensive and equitable understanding of the global impact of the COVID-19 pandemic on this caregiver population.

The findings emphasised the psychological and destabilising effects the pandemic had, impacting caregiver wellbeing as they continuously attended to their children with autism. The psychological toll was particularly notable, extending to caregivers’ quality of life and feelings of isolation (Amirova et al., 2022; Azevedo Machado et al., 2022; Dekker et al., 2022; Logrieco et al., 2022; Polónyiová et al., 2022). It was interesting to note that although the study recruited mothers and fathers, the majority of all respondents across the studies were mothers. This is a significant finding reflective of the type of available evidence about the experiences of families of children with autism. Previous research suggests that the experiences of mothers and fathers of children with autism differ, encompassing differences in quality of life, stress levels, coping strategies and the types of support needed (Grebe et al., 2022; Johnson & Simpson, 2013; Vernhet et al., 2022). Notably, in numerous African countries, the primary caregiver may not necessarily be the mother, with grandparents, particularly in South Africa, assuming a significant caregiving role (Booys et al., 2015). This highlights the importance of inclusion and focus on other caregivers and extended family members in future research.

Caregivers grappling with mental health challenges encountered obstacles in leveraging technological means to access support from friends, family and health care services. Caregivers’ mental health struggles hindered their use of technology to seek assistance, leading to reduced wellbeing as daily routines turned monotonous (Huang et al., 2021; Yılmaz et al., 2021). Proactive support and coping strategies are imperative to address these challenges. Specific resources, research and guidelines must be tailored to caregivers of children with autism. With the acknowledged risk of skill regression, caregiver burden escalated worldwide (Corbett et al., 2021; White et al., 2021). This burden emanated from grappling with structural changes, from managing behavioural issues to accommodating shifting routines and addressing service limitations while fulfilling daily tasks. Therefore, it is important to consider the ways in which caregivers should continue to be supported through access to resources, utilisation of online supportive services such as through social media platforms or online support groups, psychological and therapeutic support, and encouraging self-care.

Telehealth’s viability for non-verbal children or those with attention deficit hyperactivity disorder (ADHD) and auditory issues was questioned (Manning et al., 2021). Research has highlighted that therapy provided to children with autism and their families should be targeted to ensure that intervention will yield positive outcomes (Bundy et al., 2023). Therefore, it is imperative for researchers to delve further into devising solutions for such obstacles when caregivers employ telehealth services, such as coaching without the child present (Pacione, 2022) and the need to fully understand the necessary adjustments required in this domain for children with diverse levels of support and severity. Additionally, to consider caregivers who are situated in low-income environments and might lack access to equivalent resources and tools is essential. Alternatively, exploration of other modes of service delivery should also be considered for both caregivers and children with autism in the future.

### Strengths and limitations

The strength of this review lies in its comprehensive search strategy, which aimed to identify all published studies regardless of methodological rigour or research quality. However, some limitations need to be acknowledged. It is worth mentioning that this review predominantly incorporated research and articles primarily originating from the initial wave of the pandemic. This circumstance does restrict the breadth of our analysis. The review did not incorporate grey literature, potentially missing out on valuable unpublished or non-peer-reviewed sources, which often contain valuable insights that could have enriched the findings. Additionally, lower-impact or niche publications may have been overlooked, especially for research conducted in low- and middle-income countries. Furthermore, this review only used two reviewers and a third reviewer may have provided improved triangulation and decision making.

## Conclusion

While the pandemic brought unprecedented challenges, it also shed light on systemic issues that have long affected caregivers. This study not only enriches the discourse but also lays a foundation for meaningful improvements in the support and care for children with autism and their caregivers beyond the COVID-19 pandemic. Insights from the pandemic can guide the development of more resilient and flexible support systems that can adapt to crises. Future policies and support frameworks could benefit from incorporating the lessons learned during the pandemic, such as flexibility in service provision and the use of technology to support caregiving activities. This includes creating contingency plans for service continuity, expanding online and telehealth services, and providing targeted financial and psychological support for caregivers. Furthermore, it is worth noting that a predominant portion of study participants were mothers. This focus on mothers as participants constrains our comprehension of diverse family dynamics and the roles that fathers might play and future research should explore the experiences.

## References

[CIT0001] Adams, S.N., Seedat, J., & Neille, J. (2022). Life under lockdown for children with autism spectrum disorder: Insights from families in South Africa. *Child: Care, Health and Development*, 48(6), 1008–1016. 10.1111/CCH.12996 35253243

[CIT0002] Adams, S.N., Verachia, R., & Coutts, K. (2020). ‘A blender without the lid on’: Mealtime experiences of caregivers with a child with autism spectrum disorder in South Africa. *South African Journal of Communication Disorders*, 67(1), 1–9. 10.4102/sajcd.v67i1.708 PMC7669972 33179942

[CIT0003] Aderinto, N., Olatunji, D., & Idowu, O. (2023). Autism in Africa: Prevalence, diagnosis, treatment and the impact of social and cultural factors on families and caregivers: A review. *Annals of Medicine and Surgery*, 85(9), 4410–4416. 10.1097/MS9.0000000000001107 37663716 PMC10473371

[CIT0004] Alhuzimi, T. (2021). Stress and emotional wellbeing of parents due to change in routine for children with Autism Spectrum Disorder (ASD) at home during COVID-19 pandemic in Saudi Arabia. *Research in Developmental Disabilities*, 108, 103822. 10.1016/J.RIDD.2020.103822 33271447

[CIT0005] Al Naamani, K., AlSinani, S., & Barkun, A.N. (2020). Medical research during the COVID-19 pandemic. *World Journal of Clinical Cases*, 8(15), 3156–3163. 10.12998/WJCC.V8.I15.3156 32874970 PMC7441262

[CIT0006] Althiabi, Y. (2021). Attitude, anxiety and perceived mental health care needs among parents of children with Autism Spectrum Disorder (ASD) in Saudi Arabia during COVID-19 pandemic. *Research in Developmental Disabilities*, 111, 103873. 10.1016/j.ridd.2021.103873 33540358

[CIT0007] Amaral, D.G., & De Vries, P.J. (2020). COVID-19 and autism research: Perspectives from around the globe. *Autism Research*, 13(6), 844–869. 10.1002/AUR.2329 32592337 PMC7361219

[CIT0008] Amirova, A., CohenMiller, A., & Sandygulova, A. (2022). The effects of the COVID-19 pandemic on the well-being of children with autism spectrum disorder: Parents’ perspectives. *Frontiers in Psychiatry*, 13, 913902. 10.3389/FPSYT.2022.913902/BIBTEX 35958650 PMC9359431

[CIT0009] Arazi, A., Koller, J., Zachor, D.A., Golan, O., Sadaka, Y., Eytan, D., Stolar, O., Atzaba-Poria, N., Golan, H., Menashe, I., Meiri, G., Gabis, L.V., & Dinstein, I. (2022). Home-quarantine during the initial Covid-19 outbreak in Israel: Parent perceived impact on children with ASD. *Heliyon*, 8(6), e09681. 10.1016/j.heliyon.2022.e09681 35698655 PMC9176182

[CIT0010] Arksey, H., & O’Malley, L. (2005). Scoping studies: Towards a methodological framework. *International Journal of Social Research Methodology*, 8(1), 19–32. 10.1080/1364557032000119616

[CIT0011] Athbah, S. (2021). Covid-19 impact on children with autism spectrum disorder and intellectual disability: Study in Saudi Arabia. *Journal of Educational and Social Research*, 11(6), 78. 10.36941/jesr-2021-0130

[CIT0012] Ausderau, K., John, B.S., Kwaterski, K.N., Nieuwenhuis, B., & Bradley, E. (2019). Parents’ strategies to support mealtime participation of their children with autism spectrum disorder. *American Journal of Occupational Therapy*, 73(1), 7301205070p1. 10.5014/ajot.2019.024612 PMC6402415 30839262

[CIT0013] Azevedo Machado, B., Silva Moro, J., Massignam, C., Cardoso, M., & Bolan, M. (2022). Fear, changes in routine and dental care for children and adolescents with autism spectrum disorder in the COVID-19 pandemic: A survey with Brazilian parents. *Special Care in Dentistry*, 42(4), 352–360. 10.1111/scd.12683 34897755

[CIT0014] Baweja, R., Brown, S.L., Edwards, E.M., & Murray, M.J. (2022). COVID-19 pandemic and impact on patients with autism spectrum disorder. *Journal of Autism and Developmental Disorders*, 52(1), 473–482. 10.1007/s10803-021-04950-9 33689088 PMC7943706

[CIT0015] Bellomo, T.R., Prasad, S., Munzer, T., & Laventhal, N. (2020). The impact of the COVID-19 pandemic on children with autism spectrum disorders. *Journal of Pediatric Rehabilitation Medicine*, 13(3), 349–354. 10.3233/PRM-200740 32986631

[CIT0016] Bhat, A. (2021). Analysis of the SPARK study COVID-19 parent survey: Early impact of the pandemic on access to services, child/parent mental health, and benefits of online services. *Autism Research*, 14(11), 2454–2470. 10.1002/aur.2618 34591364 PMC8578426

[CIT0017] Booys, H.R., Adendorff, S.A., & Moodley, T. (2015). Grandparents as primary caregivers: A factor in the academic functioning and behaviour of their grandchildren. *Journal of Educational Studies*, 14(2), 139–154.

[CIT0018] Bozkus-Genc, G., & Sani-Bozkurt, S. (2022). How parents of children with autism spectrum disorder experience the COVID-19 pandemic: Perspectives and insights on the new normal. *Research in Developmental Disabilities*, 124, 104200. 10.1016/J.RIDD.2022.104200 35180544 PMC8841154

[CIT0019] Braun, V., & Clarke, V. (2006). Using thematic analysis in psychology. *Qualitative Research in Psychology*, 3(2), 77–101. 10.1191/1478088706qp063oa

[CIT0020] Bundy, R., Mandy, W., Kenny, L., & Ali, D. (2023). Autistic people and telehealth practice during the COVID-19 pandemic: A scoping review. *Review Journal of Autism and Developmental Disorders*, 1–19. 10.1007/s40489-023-00387-137363697

[CIT0021] Cassidy, S.A., Nicolaidis, C., Davies, B., Rosa, S.D.R., Eisenman, D., Onaiwu, M.G., Kapp, S.K., Kripke, C.C., Rodgers, J., & Waisman, T. (2020). An expert discussion on autism in the COVID-19 pandemic. *Autism in Adulthood*, 2(2), 106–117. 10.1089/aut.2020.29013.sjc 36601566 PMC8992856

[CIT0022] Chen, S., Chen, S., Li, X., & Ren, J. (2020). Mental health of parents of special needs children in China during the COVID-19 pandemic. *International Journal of Environmental Research and Public Health*, 17(24), 1–14. 10.3390/ijerph17249519 PMC7765938 33353165

[CIT0023] Chen, S.D., Yu, Y., Li, X.K., Chen, S.Q., & Ren, J. (2021). Parental self-efficacy and behavioral problems in children with autism during COVID-19: A moderated mediation model of parenting stress and perceived social support. *Psychology Research and Behavior Management*, 14, 1291–1301. 10.2147/PRBM.S327377 34429669 PMC8374842

[CIT0024] Chenneville, T., & Schwartz-Mette, R. (2020). Ethical considerations for psychologists in the time of COVID-19. *American Psychologist*, 75(5), 644–654. 10.1037/amp0000661 32437180

[CIT0025] Colizzi, M., Sironi, E., Antonini, F., Ciceri, M.L., Bovo, C., & Zoccante, L. (2020). Psychosocial and behavioral impact of COVID-19 in autism spectrum disorder: An online parent survey. *Brain Sciences*, 10(6), 341. 10.3390/brainsci10060341 32503172 PMC7349059

[CIT0026] Corbett, B.A., Muscatello, R.A., Klemencic, M.E., & Schwartzman, J.M. (2021). The impact of COVID-19 on stress, anxiety, and coping in youth with and without autism and their parents. *Autism Research*, 14(7), 1496–1511. 10.1002/AUR.2521 33913261 PMC8237027

[CIT0027] Cusinato, M., Iannattone, S., Spoto, A., Poli, M., Moretti, C., Gatta, M., & Miscioscia, M. (2020). Stress, resilience, and well-being in Italian children and their parents during the COVID-19 pandemic. *International Journal of Environmental Research and Public Health*, 17(22), 1–17. 10.3390/ijerph17228297 PMC7696524 33182661

[CIT0028] Dekker, L., Hooijman, L., Louwerse, A., Visser, K., Bastiaansen, D., Ten Hoopen, L., De Nijs, P., Dieleman, G., Ester, W., Van Rijen, S., Truijens, F., & Van Der Hallen, R. (2022). Impact of the COVID-19 pandemic on children and adolescents with autism spectrum disorder and their families: A mixed-methods study protocol. *BMJ Open*, 12(1), e049336. 10.1136/bmjopen-2021-049336 PMC8795917 35078834

[CIT0029] Esentürk, O.K. (2021). Parents’ perceptions on physical activity for their children with autism spectrum disorders during the novel coronavirus outbreak. *International Journal of Developmental Disabilities*, 67(6), 446–457. 10.1080/20473869.2020.1769333 34925775 PMC8676658

[CIT0030] Fong, H.X., Cornish, K., Kirk, H., Ilias, K., Shaikh, M.F., & Golden, K.J. (2021). Impact of the COVID-19 lockdown in Malaysia: An examination of the psychological well-being of parent-child dyads and child behavior in families with children on the autism spectrum. *Frontiers in Psychiatry*, 12, 733905. 10.3389/FPSYT.2021.733905/FULL 34721108 PMC8555492

[CIT0031] Friesen, K.A., Weiss, J.A., Howe, S.J., Kerns, C.M., & McMorris, C.A. (2022). Mental health and resilient coping in caregivers of autistic individuals during the COVID-19 pandemic: Findings from the families facing COVID study. *Journal of Autism and Developmental Disorders*, 52(7), 3027–3037. 10.1007/s10803-021-05177-4 34240291 PMC8265288

[CIT0032] Grebe, S.C., Mire, S.S., Kim, H., & Keller-Margulis, M.A. (2022). Comparing fathers’ and mothers’ perspectives about their child’s autism spectrum disorder. *Journal of Autism and Developmental Disorders*, 52(4), 1841–1854. 10.1007/s10803-021-05077-7 34027629

[CIT0033] Guler, J., Stewart, K.A., De Vries, P.J., Seris, N., Shabalala, N., & Franz, L. (2023). Conducting caregiver focus groups on autism in the context of an international research collaboration: Logistical and methodological lessons learned in South Africa. *Autism*, 27(3), 751–761. 10.1177/13623613221117012 35999698 PMC9947186

[CIT0034] Hammons, A.J., & Robart, R. (2021). Family food environment during the COVID-19 pandemic: A qualitative study. *Children*, 8(5), 354. 10.3390/children8050354 33946715 PMC8146061

[CIT0035] Hatch, M., & Posel, D. (2018). Who cares for children? A quantitative study of childcare in South Africa. *Development Southern Africa*, 35(2), 267–282. 10.1080/0376835X.2018.1452716

[CIT0036] Huang, S., Sun, T., Zhu, Y., Song, S., Zhang, J., Huang, L., Chen, Q., Peng, G., Zhao, D., Yu, H., & Jing, J. (2021). Impact of the covid-19 pandemic on children with ASD and their families: An online survey in China. *Psychology Research and Behavior Management*, 14, 289–297. 10.2147/PRBM.S293426 33692639 PMC7939504

[CIT0037] Iovino, E.A., Caemmerer, J., & Chafouleas, S.M. (2021). Psychological distress and burden among family caregivers of children with and without developmental disabilities six months into the COVID-19 pandemic. *Research in Developmental Disabilities*, 114, 103983. 10.1016/J.RIDD.2021.103983 33964709 PMC9758884

[CIT0038] Jeste, S., Hyde, C., Distefano, C., Halladay, A., Ray, S., Porath, M., Wilson, R.B., & Thurm, A. (2020). Changes in access to educational and healthcare services for individuals with intellectual and developmental disabilities during COVID-19 restrictions. *Journal of Intellectual Disability Research*, 64(11), 825–833. 10.1111/jir.12776 32939917

[CIT0039] Johnson, N.L., & Simpson, P.M. (2013). Lack of father involvement in research on children with autism spectrum disorder: Maternal parenting stress and family functioning. *Issues in Mental Health Nursing*, 34(4), 220–228. 10.3109/01612840.2012.745177 23566184

[CIT0040] Johnsson, G., & Bulkeley, K. (2021). Practitioner and service user perspectives on the rapid shift to teletherapy for individuals on the autism spectrum as a result of COVID-19. *International Journal of Environmental Research and Public Health*, 18(22), 11812. 10.3390/ijerph182211812 34831567 PMC8620428

[CIT0041] Kaku, S., Chandran, S., Roopa, N., Choudhary, A., Ramesh, J., Somashekariah, S., Kuduvalli, S., Rao, V., & Mysore, A. (2021). Coping with autism during lockdown period of the COVID-19 pandemic: A cross-sectional survey. *Indian Journal of Psychiatry*, 63(6), 568. 10.4103/INDIANJPSYCHIATRY.INDIANJPSYCHIATRY_344_21 35136254 PMC8793703

[CIT0042] Kalb, L.G., Badillo-Goicoechea, E., Holingue, C., Riehm, K.E., Thrul, J., Stuart, E.A., Smail, E.J., Law, K., White-Lehman, C., & Fallin, D. (2021). Psychological distress among caregivers raising a child with autism spectrum disorder during the COVID-19 pandemic. *Autism Research*, 14(10), 2183–2188. 10.1002/aur.2589 34363330 PMC8420467

[CIT0043] Khan, Y., Khan, A., El Tahir, M., Hammoudeh, S., Al Shamlawi, M., & Alabdulla, M. (2021). The impact of COVID-19 pandemic social restrictions on individuals with autism spectrum disorder and their caregivers in the Stateof Qatar: A cross-sectional. *Research in Developmental Disabilities*, 119, 104090. 10.1016/j.ridd.2021.104090 34624722 PMC8481093

[CIT0044] Laborde, D. (2020). Poverty and food insecurity could grow dramatically as COVID-19 spreads. *COVID-19 & Global Food Security*, July, 16–20. Retrieved from https://pdfs.semanticscholar.org/3495/115d4692c8caade4ac8a2adaf658d808be29.pdf

[CIT0045] Lai, W.W., & Oei, T.P.S. (2014). Coping in parents and caregivers of children with autism spectrum disorders (ASD): A review. *Review Journal of Autism and Developmental Disorders*, 1(3), 207–224. 10.1007/s40489-014-0021-x 25800867

[CIT0046] Logrieco, M.G., Casula, L., Ciuffreda, G.N., Novello, R.L., Spinelli, M., Lionetti, F., Nicolì, I., Fasolo, M., Giovanni, V., & Stefano, V. (2022). Risk and protective factors of quality of life for children with autism spectrum disorder and their families during the COVID-19 lockdown. An Italian study. *Research in Developmental Disabilities*, 120, 104130. 10.1016/j.ridd.2021.104130 34826776 PMC8602998

[CIT0047] Moher, D., Liberati, A., Tetzlaff, J., Altman, D.G., & PRISMA Group. (2009). Preferred reporting items for systematic reviews and meta-analyses: The PRISMA statement. *Annals of Internal Medicine*, 151(4), 264–269. 10.7326/0003-4819-151-4-200908180-00135 19622511

[CIT0048] Mumbardó-Adam, C., Barnet-López, S., & Balboni, G. (2021). How have youth with autism spectrum disorder managed quarantine derived from COVID-19 pandemic? An approach to families perspectives. *Research in Developmental Disabilities*, 110, 103860. 10.1016/j.ridd.2021.103860 33486395 PMC9758011

[CIT0049] Pellicano, E., Brett, S., Den Houting, J., Heyworth, M., Magiati, I., Steward, R., Urbanowicz, A., & Stears, M. (2022). COVID-19, social isolation and the mental health of autistic people and their families: A qualitative study. *Autism*, 26(4), 914–927. 10.1177/13623613211035936 34362263

[CIT0050] Polónyiová, K., Belica, I., Celušáková, H., Janšáková, K., Kopčíková, M., Szapuová, Ž., & Ostatníková, D. (2022). Comparing the impact of the first and second wave of COVID-19 lockdown on Slovak families with typically developing children and children with autism spectrum disorder. *Autism*, 26(5), 1046–1055. 10.1177/13623613211051480 34657487

[CIT0051] Pratesi, C.B., Garcia, A.B., Pratesi, R., Gandolfi, L., Hecht, M., Nakano, E.Y., & Zandonadi, R.P. (2021). Quality of life in caregivers of children and adolescents with autistic spectrum disorder: Development and validation of the questionnaire. *Brain Sciences*, 11(7), 924. 10.3390/brainsci11070924 34356158 PMC8304644

[CIT0052] Ramlagan, S., Shean, Y.L., Parker, S., Trollip, K., Davids, A., & Reddy, S.P. (2022). Pushing the boundaries: Adapting research methodology to document the COVID-19 pandemic from a socio-behavioural perspective in a low/middle level income country: The case of South Africa. *International Journal of Social esearch Methodology*, 25(3), 323–329. 10.1080/13645579.2021.1883538

[CIT0053] Schaaf, R.C., Benevides, T., Mailloux, Z., Faller, P., Hunt, J., Van Hooydonk, E., Freeman, R., Leiby, B., Sendecki, J., & Kelly, D. (2014). An intervention for sensory difficulties in children with autism: A randomized trial. *Journal of Autism and Developmental Disorders*, 44(7), 1493–1506. 10.1007/S10803-013-1983-8 24214165 PMC4057638

[CIT0054] Siracusano, M., Segatori, E., Riccioni, A., Gialloreti, L.E., Curatolo, P., & Mazzone, L. (2021). The impact of COVID-19 on the adaptive functioning, behavioral problems, and repetitive behaviors of Italian children with autism spectrum disorder: An observational study. *Children*, 8(2), 96. 10.3390/CHILDREN8020096 33540683 PMC7913091

[CIT0055] Stankovic, M., Stojanovic, A., Jelena, S., Stankovic, M., Shih, A., & Stankovic, S. (2022). The Serbian experience of challenges of parenting children with autism spectrum disorders during the COVID-19 pandemic and the state of emergency with lockdown. *European Child and Adolescent Psychiatry*, 31(4), 693–698. 10.1007/S00787-021-01917-0 34837543 PMC8626751

[CIT0056] The Lancet. (2020). Emerging understandings of 2019-nCoV. *The Lancet*, 395(10221), 311. 10.1016/S0140-6736(20)30186-0 PMC7134625 31986259

[CIT0057] Tokatly Latzer, I., Leitner, Y., & Karnieli-Miller, O. (2021). Core experiences of parents of children with autism during the COVID-19 pandemic lockdown. *Autism*, 25(4), 1047–1059. 10.1177/1362361320984317 33435701

[CIT0058] Tremblay, S., Castiglione, S., Audet, L.-A., Desmarais, M., Horace, M., & Peláez, S. (2021). Conducting qualitative research to respond to COVID-19 challenges: Reflections for the present and beyond. *International Journal of Qualitative Methods*, 20, 1–8. 10.1177/16094069211009679

[CIT0059] Vernhet, C., Michelon, C., Dellapiazza, F., Rattaz, C., Geoffray, M.M., Roeyers, H., Picot, M.-C., Baghdadli, A., & ELENA Study Group. (2022). Perceptions of parents of the impact of autism spectrum disorder on their quality of life and correlates: Comparison between mothers and fathers. *Quality of Life Research*, 31(5), 1499–1508. 10.1007/s11136-021-03045-3 34822048

[CIT0060] White, L.C., Law, J.K., Daniels, A.M., Toroney, J., Vernoia, B., Xiao, S., SPARK Consortium, Feliciano, P., & Chung, W.K. (2021). Brief report: Impact of COVID-19 on individuals with ASD and their caregivers: A perspective from the SPARK cohort. *Journal of Autism and Developmental Disorders*, 51(10), 3766–3773. 10.1007/S10803-020-04816-6/TABLES/2 33387233 PMC7775834

[CIT0061] Yarımkaya, E., & Esentürk, O.K. (2020). The novel coronavirus (COVID-19) outbreak: Physical inactivity and children with autism spectrum disorders. *Life Span and Disability*, 23(1), 133–152.

[CIT0062] Yılmaz, B., Azak, M., & Şahin, N. (2021). Mental health of parents of children with autism spectrum disorder during COVID-19 pandemic: A systematic review. *World Journal of Psychiatry*, 11(7), 388. 10.5498/WJP.V11.I7.388 34327131 PMC8311509

